# Meta-Analysis of Microdissected Breast Tumors Reveals Genes Regulated in the Stroma but Hidden in Bulk Analysis

**DOI:** 10.3390/cancers13133371

**Published:** 2021-07-05

**Authors:** Aurora Savino, Niccolò De Marzo, Paolo Provero, Valeria Poli

**Affiliations:** 1 Molecular Biotechnology Center, Department of Molecular Biotechnology and Health Sciences, University of Turin, Via Nizza 52, 10126 Turin, Italy; niccolo.demarzo@edu.unito.it; 2 Department of Neurosciences “Rita Levi Montalcini”, University of Turin, Corso Massimo D’Azeglio 52, 10126 Turin, Italy; paolo.provero@unito.it; 3 Center for Omics Sciences, Ospedale San Raffaele IRCCS, Via Olgettina 60, 20132 Milan, Italy

**Keywords:** tumor microenvironment, meta-analysis, tumor stroma, breast cancer, LCM, microdissection, transcriptomics, microarray, database

## Abstract

**Simple Summary:**

Gene expression profiling of tumors is an essential approach for the selection of biomarkers and the investigation of the molecular mechanisms of cancer, but transcriptomic results are often difficult to reproduce due to technical biases, sample heterogeneity, or small sample sizes. Combining many datasets can help to reduce artefacts and improve statistical power. Therefore, we aimed at creating a comprehensive resource of transcriptomic datasets investigating breast cancers, focusing on microdissected tumors, which enable the distinguishing of the contribution of the tumor microenvironment from that of cancer cells. We define robust lists of differentially expressed genes and describe their relationships with clinical features in each cellular compartment, identifying clinically relevant markers that can only be retrieved by measuring their expression in the sole tumor microenvironment.

**Abstract:**

Transcriptome data provide a valuable resource for the study of cancer molecular mechanisms, but technical biases, sample heterogeneity, and small sample sizes result in poorly reproducible lists of regulated genes. Additionally, the presence of multiple cellular components contributing to cancer development complicates the interpretation of bulk transcriptomic profiles. To address these issues, we collected 48 microarray datasets derived from laser capture microdissected stroma or epithelium in breast tumors and performed a meta-analysis identifying robust lists of differentially expressed genes. This was used to create a database with carefully harmonized metadata that we make freely available to the research community. As predicted, combining the results of multiple datasets improved statistical power. Moreover, the separate analysis of stroma and epithelium allowed the identification of genes with different contributions in each compartment, which would not be detected by bulk analysis due to their distinct regulation in the two compartments. Our method can be profitably used to help in the discovery of biomarkers and the identification of functionally relevant genes in both the stroma and the epithelium. This database was made to be readily accessible through a user-friendly web interface.

## 1. Introduction

High-throughput analyses of gene expression hold great promise for the identification of biomarkers of clinical status, with the potential of predicting outcome, response to therapy, or informing researchers about molecular mechanisms underpinning disease onset and progression and identifying therapeutic targets [1]. Nevertheless, lists of candidate genes obtained through transcriptome-based studies have proven difficult to reproduce [2,3,4,5,6], raising a note of caution regarding conclusions driven by single sets of experiments. Sample collection and processing methods, protocols, and platforms may impact on the resulting gene signatures, making them non-overlapping between studies [7]. Additional variability may be introduced by patient heterogeneity, which is not sufficiently represented in small samples.

To resolve these issues, the vast amount of information present in gene expression databases such as Gene Expression Omnibus, ArrayExpress, and EGA [8,9,10] can be integrated to improve the quality of gene signatures. The advantage is twofold: on one side, a larger sample size allows for the increasing of statistical power; on the other side, merging data obtained through different experimental settings facilitates the removal single-experiments biases, improving robustness [11,12]. Meta-analyses serve this scope, providing a quantitative approach to combine the results of studies investigating the same biological system [5]. Several methods based on different statistical approaches have been proposed [13,14]: (i) aggregating gene lists based on *p*-value [15]; (ii) effect size [16]; and (iii) gene rankings [17].

Meta-analyses are extremely beneficial when applied to cancer biology, an extremely prolific field that often offers tens of independent studies analyzing the same biological or clinical question in different patient cohorts. In their simplest, yet most significant, form, meta-analyses have been applied to assess the reliability of specific genes as diagnostic and prognostic markers [18,19], while whole transcriptomic datasets have been employed for the unbiased evaluation and refinement of prognostic signatures [20,21,22,23,24,25] to identify patient subgroups [26,27,28,29,30], markers of metastatic tumors [31], and markers of resistance to treatments [32].

The tumor microenvironment is an important player in determining tumor growth, disease progression, and drug resistance [33,34,35]. It is a composite environment comprising growth factors, cytokines, and cells of different origin such as fibroblasts, endothelial cells, and immune cells [36]. Each of these components can support or inhibit tumor growth and are involved in multidirectional cross-talk among each other and with tumor cells that can influence their behavior in supporting cancer. Indeed, the pro-tumoral functions of cancer associated fibroblasts (CAFs) and immune cells in the tumor microenvironment are being studied as therapeutic targets [37,38].

Understanding the biology of each component of the tumor milieu is necessary to obtain a complete picture of tumors but obtaining compartment-specific gene expression profiles is laborious, and therefore most high-throughput datasets are based on bulk tissues. Nevertheless, relying on samples composed of cell admixtures may hide cell type specific signals and may create confounding effects. For example, tumor composition due to sampling variation significantly impacts genomic data [39] and tumor subtype definition [40]. Moreover, differences in the prognostic role of the same gene when measured in different compartments have been reported [41,42,43].

To overcome these limitations, a number of approaches have been introduced: (i) laser capture microdissection (LCM) is commonly employed to separate cell compartments that are histologically well defined [44]; (ii) spatial transcriptomics has allowed for the obtaining of spatially-resolved profiles of stroma-rich and stroma-poor regions in tumor tissues [45]; (iii) single-cell techniques have allowed the distinguishing of transcriptomic profiles of different cell types within a tumor [46] and the dissection of the CAFs’ transcriptional heterogeneity [47]; (iv) computational methods have been designed to deconvolve the contribution of each cell type to the final bulk gene expression profile in silico [48]. Despite all of these methods being valuable, single-cell techniques are affected by loss of information resulting from dropouts and zero-inflation, and due to the cost, they are usually only applied to screen a few tumors in a single study, impeding the correlation of gene expression profiles with clinical features [49]. Similar problems apply to spatial transcriptomics. Deconvolution methods, on the other hand, rely on strong assumptions and depend on the quality of the specific signatures of the cell type, which are applied as input in the model. Finally, LCM, despite not allowing single-cell resolution, represents a good compromise to disentangle the specific contribution of the tumor epithelium and microenvironment and collect information on many clinically distinct samples.

Here, we performed a meta-analysis of 48 transcriptomic datasets from LCM breast tumor samples, studying the specific epithelium and stroma contribution to the gene expression profiles of bulk tumors. We identified genes robustly changing their expression in each compartment with respect to a normal breast and selected categories of genes with compartment-specific regulation and correlation with clinical features. Finally, we made the whole database and the harmonized metadata available, providing a web-based interface to facilitate its interrogation (https://aurorasavino.shinyapps.io/metalcm/, accessed on 22 June 2021).

## 2. Materials and Methods

### 2.1. Search of Datasets

Transcriptomic datasets of breast tumors analyzed in their stromal compartment were searched on Gene Expression Omnibus (GEO, https://www.ncbi.nlm.nih.gov/geo/) on 20 December 2020, using the search terms “Breast cancer” AND “lcm” or “Breast cancer” AND “stroma” or “Breast cancer” AND “microdissect*” and selecting the study type as “Expression profiling by array” and “Expression profiling by high-throughput sequencing”. They were then individually screened to discard datasets not comprising untreated tumor samples. The whole list of datasets comprised in the final database is available in Table S1. Original works describing each dataset can be found in [50,51,52,53,54,55,56,57,58,59,60,61,62,63,64,65,66,67,68,69,70,71,72,73,74,75,76,77,78,79,80,81,82,83,84,85,86,87,88,89,90,91,92,93,94].

### 2.2. Data Download and Pre-Processing

GEO datasets were downloaded using the GEOquery package [95]. Normalized (FPKM) breast cancer data from the TCGA data were obtained through TCGA biolinks [96], and METABRIC transcriptome data were obtained from synapse.org (syn2160410, accessed on 26 April 2021). Clinical and biological annotations were obtained using the same methods.

Mapping of the probes to the gene symbols was obtained from the respective platforms’ information in GEO for each dataset, and in case of multiple probes mapping to the same gene symbol, the probe with the highest mean expression across the dataset’s samples was chosen.

Whenever data were not already log transformed, we applied log2 transformation, adding an offset of 1 when the data minimum value was 0.

Replicates were merged calculating the average of their expression signals before log transformation. In GSE4823, expression values of dye-swap replicates (not log transformed) were inverted before averaging. In GSE8977, to allow variance stabilization, negative values were removed prior to log transformation.

### 2.3. Database Metadata

After defining the biological and clinical annotations to be gathered, Aurora Savino and Niccolò De Marzo independently collected them from GEO. Discrepancies were then individually checked and resolved. Moreover, for datasets GSE14548, GSE16873, GSE20437, GSE21947, GSE22513, GSE26910, GSE33692, GSE35019, GSE38959, GSE5764, and GSE72644, additional clinical annotations were obtained from the tables of the original manuscripts.

To allow for the comparison of the different datasets, annotations were harmonized as much as possible.

When multiple samples from the same subject were available and were matched to the subject of origin, all clinical annotations were assigned to all samples, including the histologically normal ones.

Details regarding single annotations are provided in Appendix A.

### 2.4. Meta-Analysis Pipeline

The meta-analysis involves the following steps: (1) define the conditions to be compared (e.g., tumor stroma vs. normal stroma); (2) select all datasets in the database comprising samples belonging to both conditions; (3) for each separate dataset, obtain the differentially expressed genes (*p*-values and logFC); (4) keep only the genes with a probe in all microarray platforms of the selected datasets; (5) collapse the *p*-values by taking the differential expression sign into account, thus obtaining a *p*-value for each gene’s up-regulation and a *p*-value for each gene’s down-regulation; (6) adjust the resulting *p*-values for multiple testing. Importantly, as stated in step 3, each dataset was analyzed separately to avoid batch effects.

The procedure for each step is detailed below (Section 2.5 and Section 2.7).

### 2.5. Differential Expression

Differential expression was calculated using limma [97] for each separate dataset, and information about the subject was added as a factor when the samples were subject-matched.

### 2.6. Datasets’ Comparison

The comparison between two different datasets was performed by considering the gene symbols represented in both arrays and computing the Spearman correlation of their logFC. For experiments using mouse systems, human orthologs were obtained from biomaRt [98]. Specifically, in Section 3.2, all datasets comprising both cancerous and non-cancerous stromal samples were used, and pairwise correlations between datasets are shown. The datasets used for each class of comparisons are invasive BC vs. normal tissue (GSE10797, GSE33692, GSE35019, GSE8977); invasive BC vs. normal counterpart (GSE14548, GSE26910, GSE35019, GSE83591, GSE90505); DCIS vs. normal tissue (GSE33692, GSE35019); DCIS vs. normal counterpart (GSE14548, GSE35019); in vitro samples derived from invasive BC vs. normal tissue (GSE20086, GSE29270); in vitro samples derived from invasive BC vs. normal counterpart (GSE29270); and in vitro samples derived from carcinomas (not invasive) vs. normal tissue (GSE129189, GSE45256). In Section 3.3 the average logFC obtained after comparing tumor vs. normal samples in either the epithelium or in the stroma is shown, with DEGs obtained as described in Section 2.8.

### 2.7. Collapsing p-Values

Uncorrected *p*-values for each gene and each dataset, obtained either from differential expression or correlation with clinical features, were collapsed with Fisher’s method [99] from the metap package (https://CRAN.R-project.org/package=metap, accessed on 26 April 2021). Two different tests were performed separately for testing coherent up- or down-regulation (positive or negative correlation) and for taking logFC signs (correlation signs) into account. *p*-values were then cut at 2.2 × 10^−16^. Since all tests were two-sided, one-sided *p*-values were obtained with the two2one function from the metap package before applying Fisher’s method. Resulting *p*-values were corrected for multiple testing with the p.adjust function from R stats with the default Holm method.

### 2.8. Definition of DEG Categories

The meta-analysis of differentially expressed genes between invasive BC and normal/normal counterpart tissues, performed separately for epithelial and stromal samples, made use of the following datasets: GSE10797, GSE33692, GSE35019, GSE8977, GSE14548, GSE26910, GSE83591, and GSE90505 for the stroma; and GSE10780, GSE10797, GSE33692, GSE38959, GSE45581, GSE14548, GSE5764, GSE72644, and GSE83591 for the epithelium. The analysis followed the steps described in Section 2.4 to obtain lists of DEGs in the two tissues. We collapsed the results obtained by comparing tumor vs. normal tissue and tumor vs. normal counterpart. One dataset, GSE35019, allowed both comparisons (tumor stroma vs. normal stroma, tumor stroma vs. stromal normal counterpart), and to satisfy Fisher’s method for the assumption of independence, we only kept the tumor vs. normal comparison for this dataset. Keeping only the genes with a probe in all selected datasets, 9523 genes were analyzed for their differential expression in the stroma, and 10,623 were analyzed in the epithelium.

To define the classes of the DEGs being regulated in both the stroma and the epithelium, or with evidence of differential expression in only one of the two tissues, collapsed and adjusted *p*-value cutoff was set to 0.05, while no evidence of differential expression called for nominal *p*-values > 0.05. Thus, for example, the genes that were significantly up-regulated in only the tumor stroma and not differentially expressed in the tumor epithelium are those with the stromal up-regulation *p*-value (adjusted) < 0.05 and both epithelial up- and down-regulation nominal *p*-values > 0.05.

### 2.9. Assessment of DEG Groups’ Robustness

Since all the available datasets were used and no other independent study was thus available, we used a cross validation procedure in order to assess the robustness of the DEGs. Specifically, we divided the datasets selected for the comparisons, indicated in Section 2.8, into 2 groups of approximately equal size (4 and 4 datasets for the stroma, 4 and 5 datasets for the epithelium). In one group made of 4 stromal datasets and 4 epithelial datasets, which was the training set, we defined the DEG classes as described. In the second group, the test set, we tested for the differential expression of genes belonging to the DEG classes according to the same criteria but using nominal *p*-values. We repeated the same procedure 100 times by randomly selecting different combinations of datasets as training and test sets.

### 2.10. Enrichment for Functional Categories

Gene ontology enrichment was calculated with the enrichGO function from the clusterProfiler package [100] using “Biological Process” GO categories and default parameters.

### 2.11. Relationship with Clinical Features

The relationship between gene expression and clinical features (grade, age at diagnosis, size) was obtained by computing Spearman’s correlation coefficients and their corresponding *p*-values with the rcorr function from the Hmisc package (https://CRAN.R-project.org/package=Hmisc, accessed on 26 April 2021).

The relationship with survival in the METABRIC cohort was determined by dividing patients in two groups by the median expression value of the gene of interest and computing their difference in disease free survival with the Kaplan–Meier method and the log-rank test through the survival package (https://CRAN.R-project.org/package=survival, accessed on 22 April 2021).

The datasets used in the analysis of the correlation between DEG groups with clinical features (Section 3.3) are listed below. Only samples annotated as invasive BC were used. Grade in stroma: GSE12622, GSE14548, GSE35019, and GSE90505; grade in epithelium: GSE1378, GSE14548, GSE35019, GSE5764, and GSE72644; age in stroma: GSE12622, GSE14548, GSE26910, GSE90505, and GSE90521; age in epithelium: GSE1378, GSE14548, GSE38959, GSE72644, and GSE13293; node in stroma: GSE12622, GSE14548, GSE90505, GSE35019; node in epithelium: GSE1378, GSE14548, GSE5764, and GSE35019; size in stroma: GSE14548, GSE35019, GSE90505, and GSE90521; and size in epithelium: GSE1378, GSE14548, GSE35019, and GSE72644. Correlation coefficients with a specific clinical feature in the selected datasets were obtained for each gene and then averaged across all datasets.

The meta-analysis of correlation with clinical features was performed as described in Section 2.4, but the computation of Spearman’s correlation and the corresponding *p*-values were completed with the rcorr function from the Hmisc package (https://CRAN.R-project.org/package=Hmisc), and these values were used in step 5 of the meta-analysis.

### 2.12. Epithelial, Stromal and Vascular Scores

Scores for epithelial, stromal, and vascular signatures expression were calculated with a single sample GSEA (ssGSEA) via the GSVA package [101] using signatures obtained from the current meta-analysis comparing tumor and normal gene expression in samples of epithelial, stromal, or vascular origin.

Stromal and epithelial markers were obtained when comparing epithelial and stromal gene expression profiles in invasive BC samples from the same dataset and merging the *p*-values with the Fisher method as described above. The datasets used are GSE10797, GSE14548, GSE33692, GSE35019, GSE41228, GSE5847, GSE59772, GSE68744, GSE81838, GSE83591, and GSE88715. To achieve a higher stringency, we only retained DEGs with |average logFC| > 1.

Multivariate Cox models were fit with the coxph function from the survival package (https://CRAN.R-project.org/package=survival).

### 2.13. Plots and Statistical Analyses

All statistical analyses were performed with R 4.0.4 [102].

Packages used for plotting are R base graphics, ggplot2 [103], ggsignif (https://CRAN.R-project.org/package=ggsignif, accessed on 10 April 2021), ggvenn (https://CRAN.R-project.org/package=ggvenn, accessed on 16 April 2021), survminer (https://CRAN.R-project.org/package=survminer, accessed on 16 April 2021), and pheatmap (https://CRAN.R-project.org/package=pheatmap, accessed on 16 April 2021).

### 2.14. Web App

The web app (https://aurorasavino.shinyapps.io/metalcm/, accessed on 22 June 2021) was built with the Shiny package (https://CRAN.R-project.org/package=shiny, accessed on 26 April 2021) taking advantage of rintrojs [104], shinybusy (https://CRAN.R-project.org/package=shinybusy, accessed on 10 May 2021), shinythemes (https://CRAN.R-project.org/package=shinythemes, accessed on 10 May 2021), and shinyWidgets (https://CRAN.R-project.org/package=shinyWidgets, accessed on 10 May 2021).

The data that can be easily interrogated with the app are datasets of primary invasive breast cancers, excluding inflammatory and micropapillary cancers. A tutorial describing tool’s usage is shown by pressing the “Tutorial” button. The user can choose between two conditions to compare based on compartment (stroma or epithelium), disease status (invasive BC, normal or normal counterpart—“counterpart”), and PAM50 subtype. The analysis pipeline applied is the same as the one used in this work and detailed above (Section 2.4). Additionally, the enrichment of user-defined gene lists for DEGs can be assessed in the second tab, displaying the result of a one-tailed Fisher test (fisher.test function from R stats). To be noted, when the user inputs a gene list, the correction for multiple testing, used to determine the list of DEGs shown in the first tab, is applied to those genes only. The *p*-adjustment is applied to all genes when obtaining DEGs for the Fisher test.

The DEG lists can be downloaded, and include collapsed *p*-values, average logFC across analysed datasets, and individual-datasets’ *p*-values and logFCs.

### 2.15. Stat3 Signatures

Signatures of Stat3 activity were obtained from Azare et al. [105], Dauer et al. [106], IL6 and Jak/STAT from MSigDB [107], Alvarez et al. [108], Tell and Horvath [109], and Sonnenblick et al. [110].

## 3. Results

### 3.1. Database Construction

We collected 48 transcriptomic datasets of breast tumors or breast hyperplasias deposited in the Gene Expression Omnibus (GEO) database, selecting experiments where different cellular compartments were separated prior to RNA extraction. Most of the datasets (43) derive from laser capture microdissected primary tumors, while a minority measure gene expression of cancer associated fibroblasts (CAFs) grown in vitro, derived from either primary tumors or from mouse models. Overall, we collected 2144 samples, 2048 of which derive from primary tumors. The complete list of datasets is available in Table S1.

To facilitate the comparability of different experiments, we mapped the probes used for each specific experiment to gene symbols, and we did an extensive and careful harmonization of biological and clinical annotations, as detailed in the Methods (Section 2.3). Specifically, we gathered information about cellular compartment, disease status, receptor status (estrogen receptor, progesteron receptor, and HER2 amplification), PAM50 subtype, tumor histology, size, grade, TNM stage as well as the overall pathological stage, node positivity, recurrence, response to treatment, and patient’s age at diagnosis, ethnicity, and menopause status (Table 1 and Table 2 and Table S2). Moreover, wherever possible and appropriate, we predicted the PAM50 breast cancer (BC) subtype from gene expression, obtaining a good concordance between subtype and receptor expression (Figure S1) and inferred clinical variables from other available clinical annotations (Section 2.3 and Appendix A). Importantly, for 11 of the 48 datasets, we found clinical annotations that were made available by the authors of the original publication but that did not accompany the corresponding dataset in GEO. Their inclusion in our database significantly improved clinical annotations, which initially were relatively scarce. For example, we increased the number of samples annotated for age from 530 to 756 and for size from 122 to 378. The complete database comprising gene expression data and metadata is available in Tables S3 and S4, while Table S2 comprises the complete summary of available clinical features.

### 3.2. Primary and In Vitro Datasets Are Only Slightly Correlated

**Figure 1:**
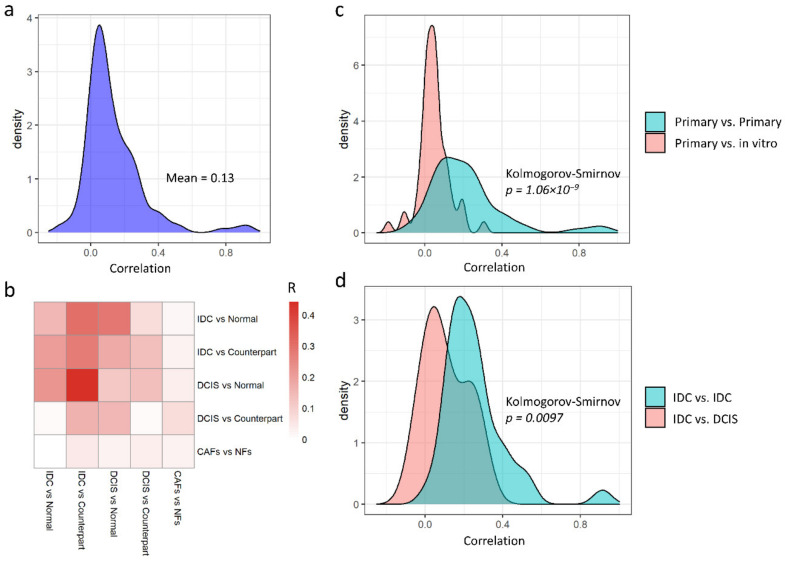
Similarity between stroma datasets of detected genes changing between normal and cancer samples. (**a**) Distribution of Pearson’s correlation between log2 fold changes (logFC) of genes between cancerous/non-cancerous conditions in each dataset, showing an average correlation of 0.13. All datasets comprising both cancerous and non-cancerous stromal samples were used, and pairwise correlations between datasets are shown. (**b**) Correlation of logFC between groups of datasets comparing invasive ductal carcinoma (IDC) and normal breast tissue from healthy donors (normal) or histologically normal tissue adjacent to tumor (counterpart), cuctal carcinoma in situ (DCIS) vs. normal or counterpart, cancer associated fibroblasts (CAFs) and normal fibroblasts (NFs) grown in vitro. Red indicates a high average positive correlation. (**c**) Distribution of Pearson’s correlations between logFC obtained from datasets sampling primary tumors or comparing logFC obtained from primary tumors or from in vitro experiments. (**d**) Distribution of Pearson’s correlations between logFC obtained from datasets sampling IDCs or comparing logFC obtained from IDCs and from DCIS.

First, we quantified gene expression changes between normal and tumor stroma in primary invasive ductal carcinomas (IDC), ductal carcinoma in situ (DCIS), or CAFs grown in vitro. As a reference condition, we used samples from cancer patients labelled as histologically normal (“normal counterpart” or simply “counterpart”), or, where available, normal breast tissue from reduction mammoplasty (“normal”). To avoid batch effects, we analyzed each dataset separately and then compared gene expression fold changes to assess similarities and differences between datasets. We were able to perform a total of 18 comparisons, as detailed in the Methods (Section 2.6). The correlations between pairs of comparisons are globally, albeit slightly, positive (Figure 1a, mean correlation of 0.13), indicating intrinsic differences between the datasets. Moreover, different classes of datasets display different degrees of similarity (Figure 1b). Indeed, for example, primary tumors and in vitro samples appeared to be only slightly positively correlated (Figure 1b,c, ρ = 0.05). Similarly, IDC and DCIS samples behave differently (Figure 1d), motivating us to keep them separated for meta-analytic purposes in order to limit the biological heterogeneity.

### 3.3. Non-Redundant Information Is Obtained by Separating Different Tissues

**Figure 2:**
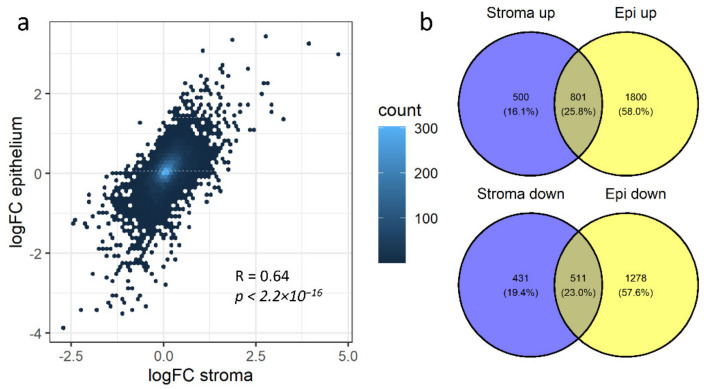
DEGs comparison between stroma and epithelium. (**a**) Cross-datasets average logFC for genes measured in normal/cancerous stroma (X axis) or in normal/cancerous epithelium (Y axis). Pearson’s correlation and *p*-value are indicated; the colour indicates the number of overlapping dots. (**b**) Venn diagrams comparing significant DEGs (*p*-adjusted < 0.05) detected in the stroma and in the epithelium.

We then took advantage of the tissue specificity of the collected datasets to compare tumor epithelium and stroma gene expression behaviors. We thus calculated differentially expressed genes (DEGs) for each dataset comprising normal and tumor samples (or normal counterpart and tumor), analyzing epithelium and stroma separately. We then collapsed the differential gene expression statistics to obtain a global measure of the reliability of the gene expression changes across all of the available datasets. As microarray platform and pre-processing can impact the measure of fold changes in differential gene expression, we chose to apply the widely employed Fisher’s method, summing the log-transformed *p*-values obtained from independent studies [99]. To limit the heterogeneity of input data, we only employed invasive BC samples for this analysis. In total, we could perform nine comparisons for epithelium and eight for stroma (datasets used are listed in the Methods, Section 2.8). Strikingly, when comparing the average fold changes for each gene in tumor stroma or epithelium, we observed that most genes behave similarly across compartments (Figure 2a), suggesting coordinated gene expression reorganization between the tumor and the surrounding cells. Alternatively, it is possible that, despite the use of LCM, the two compartments have not been perfectly separated, resulting in shared DEGs.

In the meta-analytic setting, we defined lists of robust DEGs. Specifically, we found 4390 DEGs in the epithelium (2601 up- and 1789 down-regulated in tumors) and 2243 DEGs in the stroma (1301 up- and 942 down-regulated) (Figure 2b, Table S5). The full list of fold changes and *p*-values obtained for each dataset and condition is available in Table S6, and GO categories enriched for each class are listed in Table S7. Of note, by combining the information present in several datasets, we were able to identify 229 DEGs that would not be identified in any individual dataset if analyzed separately.

Although, as mentioned above, changes in gene expression in the tumor stroma and epithelium are globally correlated (Figure 2a). For 17% of the DEGs, there was statistically significant evidence of differential expression in only the stroma/epithelium. Moreover, 104 genes showed significant differential expression in both the compartments but with opposite signs (Table S8). We posit that genes that are either regulated in one compartment only or with opposite regulation between compartments, though potentially relevant in tumor progression, may be hidden in bulk datasets due to their regulation in one compartment being confounded by a different regulation in the other.

**Figure 3:**
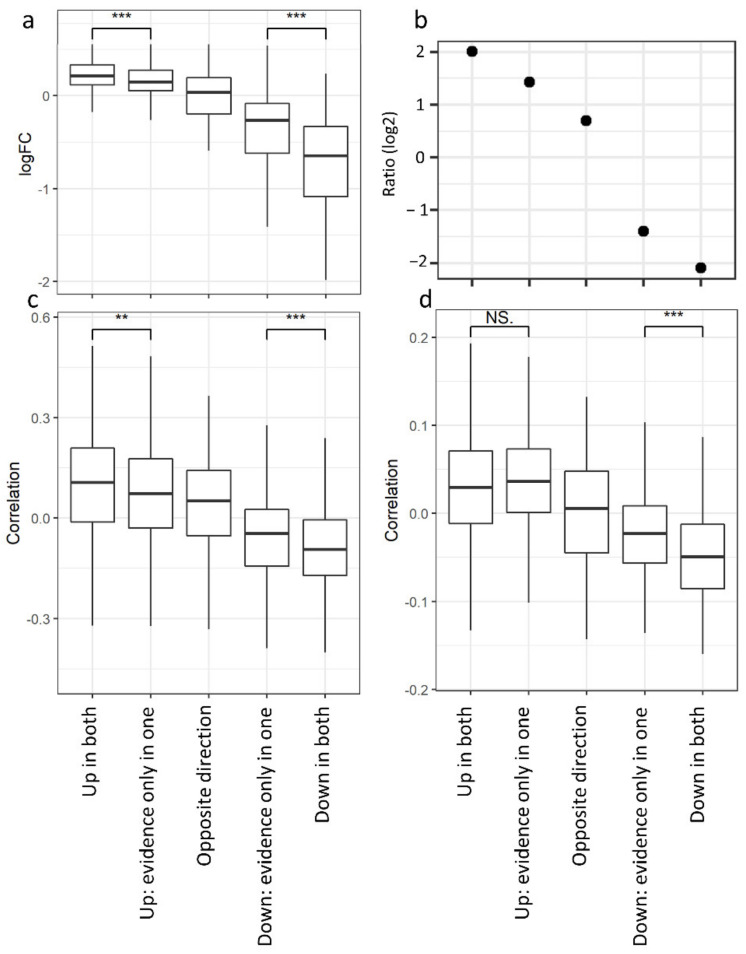
The 5 classes of DEGs in bulk samples. Genes regulated in both tumor epithelium and stroma, significantly regulated only in one of the two compartments and with opposite regulation in the two compartments show different degrees of: (**a**) differential expression between tumor and normal samples in the bulk samples of the TCGA cohort; (**b**) ratio between the number of genes significantly correlated with poor prognosis and the number of genes significantly correlated with good prognosis in the METABRIC cohort. High values indicate that many genes are correlated with poor prognosis, while inversely, negative values indicate that many genes are correlated with good prognosis; Spearman’s correlation with tumor grade (**c**) and size (**d**). Significance was assessed with the Wilcoxon test (** <0.01, *** <0.001, NS = Not Significant).

**Figure 4:**
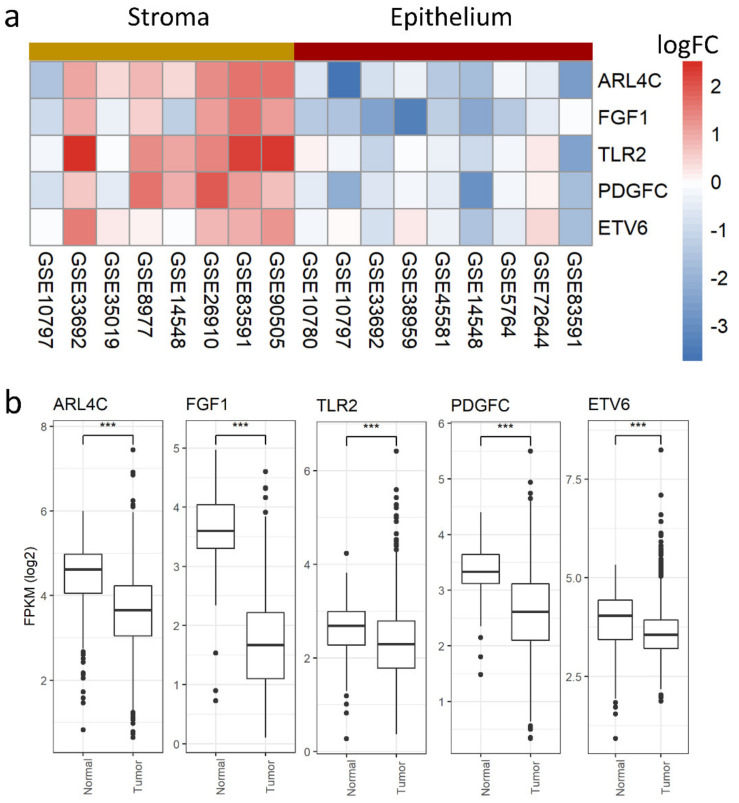
The opposite regulation of genes in tumor stroma or epithelium is hidden in bulk samples. (**a**) Top five significantly differentially expressed genes with higher expression in tumor stroma and lower expression in tumor epithelium when compared to respective compartments in normal breast tissue. Rows correspond to the five selected genes and columns to GEO IDs of datasets where the comparisons were possible and that were merged in the meta-analysis. On the left, datasets comparing normal and tumor stroma are shown, while on the right, there are datasets comparing normal and tumor epithelium. The color in the heatmap indicates logFC value for the corresponding gene and dataset. (**b**) Gene expression changes detected in bulk samples from the TCGA dataset for the five selected genes tested with the Wilcoxon test (*** *p* < 0.001).

To test this hypothesis, we selected five classes of genes: (1) genes up-regulated in both the tumor stroma and epithelium (UpBoth); (2) genes with evidence of up-regulation in only one of the two compartments (up-regulated in the epithelium—UpEpi—or in the stroma—UpStr); (3) genes with opposite signs of differential expression in the two compartments (up-regulated in the stroma and down-regulated in the epithelium—StrEpi—or down-regulated in the stroma and up-regulated in the epithelium—EpiStr); (4) genes with evidence of down-regulation in only one of the two compartments (down-regulated in the epithelium—DnEpi—or in the stroma—DnStr); and (5) genes down-regulated in both the tumor stroma and the epithelium (DnBoth) (Table S8). We tested the robustness of these classes with a 2-fold cross validation, obtaining good sensitivity and specificity (Figure S2). For each gene, we measured the average expression fold changes between normal breast and breast tumor in bulk samples obtained from the TCGA [111]. The classes with the highest fold changes are those comprising the genes that are coherently differentially expressed in both compartments, while the remaining classes show average fold changes closer to zero (Figure 3a), indicating that their genes are not detected as differentially expressed when analyzing bulk tumors. Similar results were obtained with the METABRIC dataset, which due to its extensive clinical annotations, allowed us to show graded relationships with overall patient survival and tumor grade and size in the above classes (Figure 3b–d). Thus, for two independent bulk tumor datasets, we could show that genes regulated in both tissues are more strongly differentially expressed or correlated with clinical features, suggesting the possibility that their expression is more reliably measured in bulk than genes with evidence of regulation in only one tissue or with opposite regulation in the stroma and the epithelium. Indeed, all of the classes of genes that we defined show a good robustness (Figure S2) and are therefore likely regulated but missed when analyzing bulk data. A more detailed picture of the DEGs classes indicates that genes with opposite regulation in the stroma/epithelium show correlation with the clinical features in line with their regulation in the epithelium: The EpiStr class increases its expression in bulk tumors when compared to normal breast (Figure S3a). It is enriched with genes correlated with poor prognosis (Figure S3b) and correlates with higher tumor grade (Figure S3c) and size (Figure S3d). The class of genes down-regulated in the epithelium and up-regulated in the stroma shows the opposite trend for its expression in bulk tumors and its correlation with size, though it is not related with patient survival or tumor grade (Figure S3). These observations fit with more epithelium content than stromal content in bulk samples, which hides the signal originating from the tumor stroma. Indeed, the top DEGs in the category of genes that are higher in the tumor stroma and lower in the tumor epithelium are relatively coherently up-regulated in tumor stroma across the available datasets (Figure 4a). However, they all appear significantly down-regulated in tumor samples from the TCGA (Figure 4b), confirming that their regulation in the stroma is not detected in bulk.

**Figure 5:**
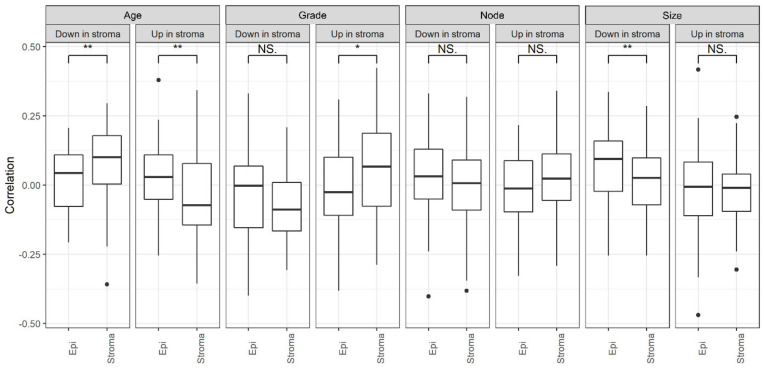
Compartment-specific relationship with clinical features for genes regulated in tumor stroma. Spearman’s correlation between gene expression and age at onset, tumor grade, lymph node status, and size for the classes of DEGs regulated only in tumor stroma, calculated for each dataset separately and then averaged. The correlation was computed when their expression was measured directly in the stroma or in the epithelium and was compared with the Wilcoxon test (* <0.05, ** <0.01, NS = Not Significant).

To test the potential relevance of genes that are differentially expressed in one compartment only as compartment-specific clinical markers, we computed their correlation with the tumor grade or age at onset when measured in the stroma or in the epithelium of invasive BC, identifying a differential correlation in line with the compartment in which each gene class is regulated (datasets and analysis are described in the Methods, Section 2.11). Indeed, genes that are up-regulated in the stroma are more strongly correlated with a higher grade and earlier onset when measured in the stroma than in the epithelium, while genes that are down-regulated in the stroma show the opposite trend (Figure 5), supporting the hypothesis of their potential compartment-specific clinical relevance, which cannot be assessed without separating them. Similar trends are observed with node positivity and size, even if some tests do not reach statistical significance (Figure 5). Nevertheless, we did not observe this consistent relationship for genes that were regulated in the epithelium (Figure S4a). Accordingly, the classes of genes with opposite regulation in the stroma and the epithelium display an opposite relationship with clinical features when measured in either the stroma or in the epithelium (Figure S4b), confirming that these classes of DEGs might also be relevant for tumor progression but differentially regulated in the two compartments.

**Figure 6:**
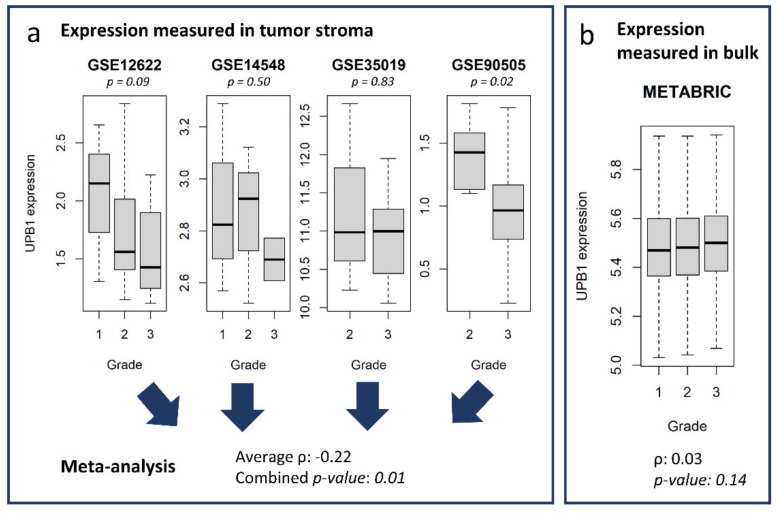
UPB1 is correlated with lower grade in the stroma, but not in bulk. (**a**) UPB1 expression in the 4 datasets with annotation for grade in the tumor stroma. The *p*-value for the correlation between UPB1 expression and tumor grade is indicated above each boxplot. Collapsing *p*-values with a meta-analysis, the statistical power increases, and the *p*-value reaches significance. Overall, the correlation between UPB1 expression and tumor grade in stroma is significantly negative. (**b**) no significant relationship between UPB1 expression and tumor grade in bulk in the METABRIC cohort.

We further showed that with our compartment-specific gene expression database and the use of meta-analysis, we can identify relationships between genes and clinical features that could not be identified otherwise. As an example, we took the 50 most down-regulated genes in tumor stroma vs. normal stroma (DnStr class). Twelve of these genes were also significantly negatively correlated with tumor grade when measured in the stroma, and most of them (75%) displayed a stronger relationship with grade in the LCM data than in bulk. We identified UPB1 (beta-ureidopropionase 1) as negatively correlated with grade in the tumor stroma but not in bulk (METABRIC dataset, Figure 6). Of note, despite the relatively small number of samples annotated for clinical features and the high within- and between-dataset variability, we were able to improve statistical power by combining multiple datasets (Figure 6a). In one case (the HSD11B2 gene, hydroxysteroid 11-beta dehydrogenase 2), the correlation in the stroma and in bulk shows opposite trends (Figure S5). This could be due to the confounding effect of multiple cell types present in mixed samples. Conversely, we identified NECAP2 (NECAP endocytosis associated 2) as up-regulated in tumor stroma and correlated with higher tumor grade when measured directly in the stroma (*p*-value = 0.01, average ρ = 0.30) but only slightly when measured in bulk (*p*-value = 0.03, ρ = 0.05).

Considering the enrichment for gene ontology categories, genes in the UpBoth class show enrichment for mitochondrion-related categories, the extracellular matrix (ECM), and antigen processing, while UpEpi genes are enriched for cell cycle and DNA repair. No categories are enriched for UpStr genes. Counter-intuitively, DnBoth genes are enriched for angiogenesis-related categories. DnEpi genes belong to the “cornification” category, while no biological processes are over-represented in DnStr genes. Interestingly, genes with opposite regulation and over-expressed in tumor stroma are enriched for cytokine secretion and Toll-like receptor 2 signaling. Full GO lists are available in Table S9.

We repeated the meta-analysis to identify robust DEGs in tumor blood vessels (datasets used: GSE15363, GSE31138, GSE7413, and GSE43379), obtaining 13 up-regulated genes and 1 down-regulated gene (Table S5).

**Figure 7:**
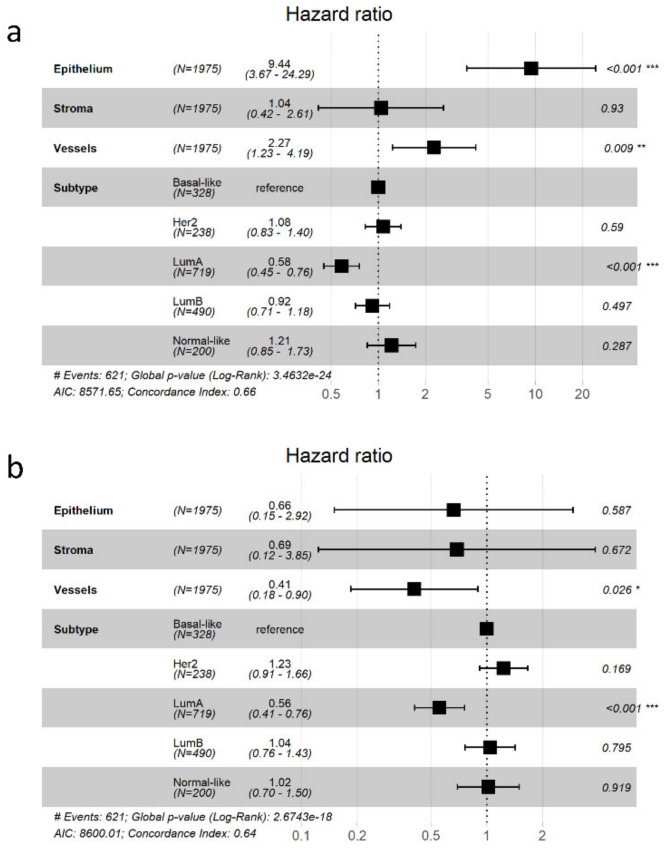
Survival models with epithelial, stromal, and vessel DEGs. Forest plots of multivariate Cox models of disease-free survival with PAM50 subtypes and expression levels of genes in gene signatures (**a**) up- or (**b**) down-regulated in tumor epithelium, stroma, or vessels when compared to corresponding normal tissues (* <0.05, ** <0.01, *** <0.001).

Despite the difficulty of accurately detecting the signal deriving from specific cellular compartment in bulk, as discussed above, we also showed in bulk samples that the epithelial and vascular signatures are independent predictors of a patient’s disease-free survival (DFS) (Figure 7). This, again, points to the relevance of cell type specific signatures in describing tumor biology. Indeed, higher expression of up-regulated genes in the epithelium or in the blood vessels independently correlate with poor prognosis in the METABRIC BC cohort (Figure 7a), while down-regulated genes in the blood vessels define the only significant signature of good prognosis (Figure 7b). We could not detect any relationship between the stromal signatures and patient DFS. This result can be attributed to the presence of multiple cell types in bulk samples, confounding compartment-specific signals. Nevertheless, it is also possible that the stromal signatures we defined, despite being correlated with tumor grade and age at onset, are not correlated with patient survival.

### 3.4. Stromal and Epithelial Markers to Impute Cell Proportions from Bulk Samples

**Figure 8:**
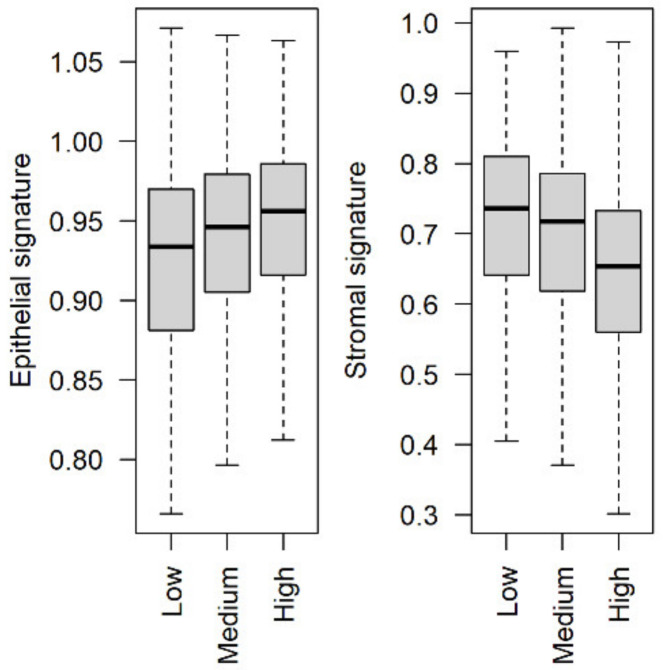
Correlation between epithelial or stromal markers and cellularity in the METABRIC cohort. Epithelial and stromal signatures were obtained through a meta-analysis comparing the expression patterns of tumor stroma and epithelium. The signatures were then used to build stromal and epithelial scores representing cell proportions for each tumor sample in the METABRIC cohort and correlated with clinician annotation of cellularity.

Computational methods to estimate cell type proportions in bulk transcriptomes often require gene expression signatures of the cell types of interest. Amongst the first proposed and most commonly applied methods is ESTIMATE [112], which based on a single sample GSEA (ssGSEA) of stromal and immune signatures to infer their proportions from the transcriptomes of cell admixtures. We computed ssGSEA on primary BC transcriptomes of the METABRIC cohort based on the stromal and epithelial markers obtained through a meta-analysis comparing tumor epithelium and stroma from our gene expression database (datasets detailed in the methods). We observed that the epithelial signature is positively correlated with the clinician-defined measure of cellularity (Spearman’s rho = 0.12, *p*-value = 2.8 × 10^−7^), while the stromal signature is negatively correlated with it (Spearman’s rho = −0.25, *p*-value < 2.2 × 10^−16^) (Figure 8), indicating that these marker lists are representative of the corresponding cell compartment and are appropriate to infer cell proportions. Moreover, our stromal signature shows a stronger correlation with cellularity than the estimated tumor purity obtained with the original ESTIMATE signatures (for which Spearman’s rho = −0.15, *p*-value = 1.4 × 10^−11^).

### 3.5. Potential Use of the Database and Web Platform

Due to the number of datasets and annotation categories, our meta-analysis offers many combinations of variables that can be selected for comparison to address specific questions. In order to make this resource available to the scientific community and to facilitate the choice of the datasets to analyze, we indicated the annotations available for each dataset (Figures S6 and S7) and some of the comparisons that can be made using a sample from a specific dataset (Figures S8 and S9). For example, there are 10 different datasets with estrogen receptor status annotations in stroma samples (Figure S6), three datasets that can be used to compare the stroma of IDC and DCIS (Figure S8b), and three datasets allowing for the comparison of Basal and LumA stroma in invasive BC (Figure S9a).

To ease the interrogation of the whole collection of datasets (available in Tables S3 and S4), we created a web app that provides a user-friendly interface, which allows the generating of lists of DEGs between two conditions and the testing of enrichment for user-provided gene lists (https://aurorasavino.shinyapps.io/metalcm/). As a use-case example, we tested the enrichment of up-regulated genes in the tumor stroma for five signatures of the pro-oncogenic transcription factor signal transducer and activator of transcription (STAT) 3 activation. We set the parameters for comparing tumor stroma and normal stroma of any BC subtype, with a *p*-value threshold of 0.05. We then loaded the gene lists corresponding to the five signatures one at a time and obtained the Fisher test enrichment *p*-value seen in the “Enrichment” Table We observed that the enrichment is significant for 3/5 of the signatures when tested in the stroma, while none show enrichment for up-regulated genes in the tumor epithelium. This observation is suggestive of the different roles of STAT3 in the two compartments, linking tumorigenesis and its up-regulation in the stroma. A lack of significance in the epithelium due to higher variability in the available datasets cannot be excluded, but it is unlikely given that the number of DEGs detected in tumor epithelium was higher than in tumor stroma (Figure 2b).

## 4. Discussion

Breast cancer is a heterogeneous disease with several cellular components playing specific roles in its development and clinical course. In particular, the microenvironment has been shown to either counteract or promote tumor progression depending on the specific conditions, and important players such as immune cells or cancer associated fibroblasts are the objects of intense study. Importantly, the analysis of bulk tumor samples comprising cell admixtures complicates disentangling the specific behaviors of each cell component. Laser capture microdissection can help in separating the contribution of different cell compartments and can still present some relevant advantages with respect to single cell techniques. Indeed, cell type separation does not rely on set of markers, but it is directly based on histological features. Moreover, the relatively contained cost allows for sampling ranges of tumors with different characteristics and the assessment of the relationships between gene expression and clinical features.

As an additional complication, breast cancer heterogeneity is hardly captured in the small sample sizes of most microarray studies, but the strong research effort dedicated to this biological system has led to a rich collection of independent datasets that can be combined to improve robustness and statistical power.

Here, we gathered 48 transcriptomic datasets of microdissected breast tumors where the stroma and the epithelium were separated prior to RNA extraction to study the distinct behavior of different cellular compartments. We carefully collected and harmonized corresponding biological and clinical annotations to facilitate data integration.

With this tool in hand, we identified genes robustly and coherently changing their expression in breast tumors when compared to normal tissue, either in the stroma or in the epithelium. Analyzing these lists separately, we detected increased expression of cell cycle related genes in the tumor epithelium and of immune-related categories in the tumor stroma. Additionally, we detected the over-expression of the non-canonical Wnt/PCP pathway, which is involved in breast cancer progression [113], synergizing with the STAT3 pathway, contributing to its aggressiveness [114] in both the stroma and the epithelium. Moreover, we observed a decrease in lipid catabolism in the tumor stroma, consistent with a potential metabolic coupling between cancer cells and the microenvironment, with stromal components reducing their consumption to release lipids and feeding cancer cell growth [115].

Comparing differentially expressed genes in the two compartments, genes that are up-regulated in both are enriched in mitochondrial-related and extracellular matrix gene ontology categories. Indeed, the extracellular matrix can act as a reservoir of growth factors, and its remodeling has been associated with metastatic spread [116]. The role of high oxidative phosphorylation in tumors is being increasingly recognized [49,117]. Counter-intuitively, down-regulated genes are enriched for angiogenesis. A similar trend had already been observed [118] and could be explained by the higher resistance of tumor cells to apoptosis under hypoxic conditions, especially in advanced tumors that also show lower microvessel densities than normal tissues [119].

Moreover, genes that are significantly regulated in only one compartment can exhibit a corresponding compartment-specific relationship with clinical features. For example, genes that are only up-regulated in tumor stroma correlate with a higher grade and earlier age at onset when measured directly in the stroma, while their relationship with clinical features is weaker when their expression is measured in the epithelium, highlighting the specific role of the stromal tissue. At the top of the list were UPB1 and HSD11B2. The latter is the enzyme that converts cortisol in cortisone, which is down-regulated in tumor stroma and correlates with a lower grade. Its decrease across tumor progression might be responsible for high cortisol levels, which have been associated with higher severity and mortality [120]. To our knowledge, this is the first time that such a relationship has been detected in the stroma. UPB1 encodes for the last enzyme in the pyrimidine degradation pathway, and its down-regulation might lead to dihydropyrimidine accumulation, linked with EMT [121]. Similarly, NECAP2 is among the top up-regulated genes in tumor stroma only, correlating with a higher grade. NECAP2 is involved in endocytic recycling [122], suggesting a potential role in regulating surface protein localization and cell-cell communication. Of note, the compartment-specific regulation of a number of genes exhibiting opposite regulation in tumor epithelium or stroma would have been missed based on bulk data: genes that are up-regulated in tumor stroma and down-regulated in tumor epithelium are enriched for cytokine secretion and Toll-like receptor 2 signaling, suggesting that the microenvironment produces different sets of cytokines than the tumor itself, possibly reflecting different chemoattraction and hence different immune cell proportions depending on tumor proximity. Indeed, immune cell distribution has been found to be of clinical relevance in cancer [123,124,125].

To the best of our knowledge, this is the first meta-analysis performed specifically on LCM transcriptomic data. We show how different platforms can be successfully integrated to reveal robust differential expression patterns and to increase statistical power, identifying differentially expressed genes that would not be identified otherwise. We note, however, that some of our results need to be cautiously interpreted given that the definition of non-differential genes cannot be given in a statistically rigorous way. Nevertheless, this caveat only applies to a limited part of our work, and the lists of DEGs in tumor epithelium, stroma, and vessels that we provide are indeed robust.

From the analysis of our database, we can conclude that although the behavior of the epithelium and the stroma at the gene expression level is globally similar, separating compartments allows for the identification of gene regulation patterns that could not be detected in bulk. Moreover, integrating many different datasets allowed us to improve statistical power and, despite the small sample size of each dataset, to identify the genes that are correlated with clinical features in a compartment-specific manner.

We showed additional use-cases of our database, such as the selection of epithelial and stromal markers that improves the correlation with cellularity compared to published signatures. Indeed, this refined signature could prove to be profitable in estimating the tumor/stroma composition of bulk tumors through deconvolution methods. Moreover, our analysis of STAT3 signaling pathway regulation revealed a particularly relevant role in tumor stroma compared to the epithelial counterpart, despite STAT3 being considered as an oncogene in many tumor types, including breast cancer [126], and was consistent with data showing an opposite role in the two compartments in colorectal cancer [127]. Specific questions can be addressed by performing the wide variety of comparisons allowed by the conditions represented in our database. A particularly interesting application will be the construction of compartment-specific gene regulatory networks. Indeed, cancer gene networks built from bulk transcriptome data are affected by the presence of multiple cell types and often include microenvironment-related gene sub-networks, confounding the identification of cancer cell gene interactions [128]. Therefore, the use of the LCM data collected here will be a valuable resource to build more specific and robust gene co-expression networks.

An important practical corollary of our work is the availability of our database as a resource for other researchers to explore via a simple web platform that allows differential gene expression and enrichment analyses (https://aurorasavino.shinyapps.io/metalcm/).

## 5. Conclusions

By collecting and harmonizing multiple datasets of LCM breast tumors, we generated a resource that can be profitably used to discover biomarkers, investigate cancer molecular mechanisms, or test specific research-driven hypotheses in a robust setting. We envision several applications for our database, from the meta-analytic comparison of the several biological conditions and clinical statuses there annotated to the construction of compartment-specific co-expression networks, which will hopefully help in the formulation of robust and specific research hypotheses.

## Figures and Tables

**Table 1:** Summary of sample features: number of samples derived from primary tumors or in vitro systems and biological/clinical annotations of primary tumor samples. Only the main levels of each factor are shown. For a complete list, refer to Table S2.

Category	# of Samples	# of Datasets
System		
Primary tumor	2048	43
In vitro	96	5
Compartment		
Epithelium	1230	32
Stroma	664	21
Vessels	64	4
Adipose	16	1
Disease status		
Invasive BC	990	31
Tumor (other)	296	11
Normal counterpart	370	17
Normal	326	16
ER status		
Positive	502	20
Negative	419	23
PR status		
Positive	306	16
Negative	435	21
Her2 status		
Positive	302	18
Negative	661	24
LN positivity		
Positive	309	15
Negative	228	17
PAM50 subtype		
Basal-like	120	16
Her2+	71	13
LumA	194	16
LumB	140	16
Normal-like	35	10

**Table 2:** Summary of clinical features for primary tumor samples (complete list in Table S2).

Age	
Median (range)	55 (27–94)
# annotated samples	756
# of datasets	18
**Size** (in mm)	
Median (range)	24 (4–161)
# annotated samples	378
# of datasets	9
**Grade**	**# of samples**
I	50
II	189
III	312
# of datasets	12

## Data Availability

All the data used in this work are available in the Supplementary Materials and easily accessible through the Shiny web app (https://aurorasavino.shinyapps.io/metalcm/, last accessed on 22 June 2021). The main functions used for data analyses can be retrieved from the Shiny app’s code.

## Supplementary Materials

The following are available online at: https://zenodo.org/record/5013252#.YNHsMegzZPY (Doi:10.5281/zenodo.5013252, last access 22 June 2021). Figure S1: Marker expression for breast cancer PAM50 subtypes, Figure S2: Sensitivity and specificity obtained in a cross validation of the five DEGs classes to assess their robustness, Figure S3: The 8 classes of DEGs in bulk samples, Figure S4: Compartment-specific relationship with clinical features for genes regulated in tumor epithelium or with opposite regulation in stroma and epithelium, Figure S5: HSD11B2 is correlated with a lower grade in the stroma but not in bulk, Figure S6: Summary of annotations available for each dataset, Figure S7: Summary of histological types available for each dataset, Figure S8: Summary of possible comparisons between disease status and histological types that can be performed in each dataset, Figure S9: Summary of possible comparisons between PAM50 subtypes that can be performed in each dataset, Table S1: List of all datasets comprised in the database, Table S2: Statistics for the annotations of primary tumors in the database, Table S3: Database metadata, Table S4: Database expression data, Table S5: Differentially expressed genes between normal and invasive BC, Table S6: Full list of genes measured across all compared datasets, Table S7: Gene ontology categories enriched in each DEG list, Table S8: Genes belonging to the 8 classes of DEGs, Table S9: Gene ontology categories enriched in each DEG class.

## Appendix A

### Appendix A.1. Details Regarding Single Annotations and Their Harmonization Compartment

The compartment column refers to the tissue of origin and can have values: “Epi” (epithelium), “Stroma”, “Vessel” (microvessels), and “Adipose”. The additional values “fine needle” and “core needle” refer to fine/core needle biopsies from the GSE32518 dataset, stroma-poor and stroma-rich, respectively.

### Appendix A.2. Disease Status

The “diseaseStatus” column indicates whether the sample has a cancerous origin. Where not otherwise specified, malignant samples were annotated as “InvasiveBC” (invasive breast cancer). “Tumor” refers to malignancies and hyperplasias annotated as DCIS (ductal carcinoma in situ), inflammatory breast cancer (IBC), IMC (invasive micropapillary carcinoma), PABC (pregnancy associated breast cancer), AH (atypical hyperplasia), and hyperplasia not otherwise specified. “Normal” indicates histologically normal breast samples from cancer-free subjects, while “counterpart” indicates histologically normal breast samples close to the tumor. Additional annotations are related to specific datasets with particular sample types: in GSE72644, multiple normal samples were obtained from along the duct leading to the tumor at different distances (annotated as “counterpart” in the case of “normal same duct” samples and as “counterpart-adjacent” in the case of “adjacent to tumor” samples) or from contralateral duct (annotated as “contralateral”); in GSE13293, benign breast disease samples with no sign of hyperplasia or atypia were collected and were denoted as “benign”; in GSE20437, histologically normal breast samples from women at high risk of breast cancer were collected and denoted as “prophylactic”; in GSE141828, histologically normal samples from women that developed cancer later were denoted as “susceptible”. These additional levels of disease status were kept separate from other normal samples in the meta-analysis because they were considered to be essentially different.

### Appendix A.3. Receptor Status (ER, PR, Her2)

Can either be “positive” or “negative”. Where the percentage of cells positive with IHC staining was indicated, “positive” was assigned to values ≥ 10%.

For the Her2 status, values of 1+ were considered “negative”, 3+ as “positive”, and 2+ as ambiguous and were converted to “positive” only in case of FISH positivity and as a missing value otherwise [129].

Samples annotated as triple negative were assigned a “negative” value to all three receptors’ statuses.

### Appendix A.4. TNBC Status

For some datasets, samples belonging to the triple negative breast cancer (TNBC) subtype was reported. Hence, in this column “yes” indicates that the sample is triple negative, and “no” indicates that the sample is not triple negative. Additionally, TNBC status was inferred from the receptor status annotations: “no” when either one of the three receptors (ER, PR, Her2) had a “positive” value and “yes” when all three receptors had a “negative” value.

### Appendix A.5. PAM50

PAM50 molecular subtype. Where this information was missing and epithelial invasive BC samples were available, the PAM50 subtype was imputed from gene expression using the genefu package [130] with the model “pam50”. PAM50 was not imputed for datasets homogeneous for the receptor status or annotated as TNBC, since the algorithm only accurately infers the subtype from sets of samples comprising a mixture of all subtypes.

### Appendix A.6. Age

Denotes patient age at onset in years.

### Appendix A.7. Grade

Histological tumor grade. Values of I, II, and III were converted to 1, 2, and 3 to facilitate computations. Similarly, “low”, “intermediate”, and “high” values were converted to 1, 2, and 3, respectively.

### Appendix A.8. Size

Tumor size in millimetres. Where multiple sizes were indicated, the highest value was kept.

### Appendix A.9. Node

Can either be “Positive” or “Negative”. Where the number of positive lymph nodes was indicated, “Positive” was assigned to values > 0.

### Appendix A.10. Stage

Overall stage. Obtained from TNM scores, and some missing values were inferred from these scores: M1 corresponds to stage IV; T1, N0, and M0 correspond to stage I; N3 and M0 correspond to stage IIIC; T4, M0, but not N3 correspond to stage IIIB.

### Appendix A.11. Tscore, Nscore, Mscore

TNM staging system [131]. Values of T and N are in part redundant with other clinical annotations but cannot be biunivocally matched, and different datasets provided different clinical categories. Hence, they have been retained and used to infer other clinical variables or viceversa. Lymph node positivity is reflected in the N score: samples annotated as node negative were then also annotated as N0, while samples annotated as N0 were also added the “negative” value in the node column. Samples with N1-N3 were annotated as node “positive”. Finally, some missing values were inferred from the overall stage with the conversion rules listed above.

### Appendix A.12. Disease Free Survival

Two columns are related to disease free survival (DFS): DFSevent indicates presence (“yes”) or absence (“no”) of relapse; DFStime indicates the time (in months) before the relapse or the follow-up time in case of no relapse.

### Appendix A.13. Histology

Contains information about the specific histological type of the tumor. IDC (invasive ductal carcinoma), ILC (invasive lobular carcinoma), DCIS (ductal carcinoma in Situ), inflammatory breast cancer (IBC), IMC (invasive micropapillary carcinoma), PABC (pregnancy associated breast cancer), AH (atypical hyperplasia), and hyperplasia not otherwise specified. Where two histological types were reported for the same sample, the most aggressive was retained (e.g., IDC+DCIS resulted in IDC annotation); the normal counterpart was annotated with the same histological type as the corresponding tumor.

### Appendix A.14. Matching

Unique ID to allow the matching of samples belonging to the same subject.

No assumptions or changes were needed for vital status, parity, menopause, treatment, response to treatment, and ethnicity annotations.
